# The Slaughter of the Innocents*Read before Tri-State Medical Society of Alabama, Georgia, and Tennessee, October, 1894.

**Published:** 1894-10

**Authors:** E. Van Goidtsnoven

**Affiliations:** Atlanta, Ga.


### THE SLAUGHTER OF THE INNOCENTS.*

BY DR. E. VAN GOIDTSNOVEN, ATLANTA, GA.

We are constantly reminded that all living things on this our planet are subject to the laws of death ; that this earth is an immense crematory; that we live upon the ashes of the dead ; that life within us presides over the perpetual conflict between anabolic and katabolic agencies ; and that metabolism is the *ultima ratio* of all disintegration, of all oxidation; that molecular death is incessantly wrought throughout our economy; that death of the individual is

* Read before Tri-State Medical Society of Alabama, Georgia, and Tennessee, October, 1894

the cessation or annihilation of all vital functions, leaving the body to decay and to dust.

Aptly does Bryant sing:

> " My heart is awed within me when I think
> Of the great miracle that still goes on,
> In silence around me, the perpetual work
> Of thy creation, finished, yet renewed
> Forever. "

The majority of mankind have from the remotest antiquity believed in a spiritual entity in every person which lives, feels, thinks, wills, and never dies, hence the sense of immortality which presupposes the existence of a soul. So awed were the ancients by overwhelming proofs of psychical agencies, that a philosophical school sprang in their midst advocating the doctrine that there is a fluid diffused throughout all nature, animating equally all living and organized beings, and that the difference which appears in their actions comes of their particular organization. This doctrine is still held by the followers of Descartes and by the new Platonists of to-day.

The Bible teaches us that " the *soul* or *anima* is coeval with life or *vita.*

"The Lord God breathed into his nostrils the breath of life and man became a living soul. Gen. 11:7.

Thus says Gaynor:

" Adam received a soul *before he breathed,*—that he might breathe, to enable him to live and breathe; a soul that originated a life in him."

"Soul is the first act of the physical organic body having *life by power.*"

Conception is but the reiteration through human agency of the first act of human creation. A human embryo is not a thing, but a person; it has already an independent vital principle, a human and immortal soul. However undeveloped this person may be, it has at every stage of its life, natural inborn and inalienable rights, even though it cannot exercise or enforce them, and he or she who deliberately sets about to destroy this life by drugs, instruments, or patent contrivances incurs the guilt of *homicide.*

Human reason has coined no certain word expressive of the creature's right in bringing death into the world, yet it plainly points the Creator of Life as the Author of death. Life is a God-given principle; God made man to his own image; man has no inherent power or authority to destroy that which he cannot create.

There are but two cases in which there is an express divine permission of putting a person to death: 1st, In case of a just condemnation for a capital crime. 2d, In case of legitimate defence. Hence the command "Thou shalt not kill" binds the conscience of the scientist as well as that of any other creature. The punishment which God inflicted upon Onan should convince any one of the infinite value which God sets upon human life and human soul.

"He spilled his seed upon the ground *lest children should be born*, and therefore the Lord slew him because he did a detestable thing."

This terrible visitation not only imparts a positive and solemn warning, which should be heeded by those who practice and recommend means to prevent conception, but ought to impress one most forcibly of the inviolability of the embryonic life.

If it is a detestable thing to prevent conception, *a fortiori* is it so to destroy that which has been already conceived, and the Onanist, the abortionist, the cephalotripsist, morally fall under the same ban.

It does not invalidate this argument to say that the mother's life is in danger, and will perish unless the fetus be removed or craniotomy be performed. The child has a right to be born. He is not an aggressor. He took no part in the sexual congress that resulted in his existence. There is no law, says a distinguished moralist, which compels a woman to have children unless she submits herself to the process by which they are engendered.

Whether this sexual congress was licit or not, the embryo is a *de jure* as well as a *de facto* issue of a concurred act, and the legitimate dweller of the precincts which God and Nature have provided for him; he is therefore neither an invader nor an intruder.

The responsibility of his *fiat* and subsequent *ego* reverts to or rests with the authors of his conception.

There is now a practice so much in vogue, that it may be well termed a national vice; so common, that it is now openly acknowledged by its perpetrators, for the commission of which the physician is even eulogized by his patient and applauded by his friends and colleagues. *It is artificial abortion.*

As Swayne says: "Husbands seek it for their wives; libertines ask it for their mistresses; seducers seek it for the unhappy victims of their licentious passions; wives,—aye! mothers even, beg it for themselves; and physicians there are who advise it and practice it not only as a *dernier ressort*, but as an expeditious means of relieving nausea and vomiting in pregnancy, and therefore call it justifiable."

If the above biblical quotations do not afford a moral lesson, and should not restrain a medical man from destroying what God has created to his own image, then religion is a farce, and it can be truly said of us, that we are licensed to kill.

I am aware that civil law indorses this practice based upon some absurd discrimination between viability and non-viability, between life and animation; but I am also aware that there are many things that are legally right and morally wrong. We all admit that civil law often fails to be the exponent of a moral equation between *meum* and *tuum.*

It is subject to mutations and frequent repeals, for to err is human, even in law. Moral law never changes; it is eternal, inviolable.

To contend that there is no soul in an embryo until a certain period of gestation, or its full delivery, is as absurd as it would be to deny life in a new grain of wheat, or in a fresh ovum.

An embryonic soul with its latent powers is as natural, and therefore as comprehensible an entity as an embryonic life with all its latent energies. Both are subject to evolution and development. We are yet to learn the *modus operandi*, or rather the acrobatic performance whereby a soul can penetrate a fetus at any stage of its development. Conception is all sufficient, and a repeated activity on the part of the creator can only exist in the imagination of those who seek a vindication in the assertion that fetal destruction is an evil perhaps, but in certain cases is lawful.

It would be inhuman and cruel to stand by and see a woman die of exhaustion. It would be murder to kill an innocent child that we might save a woman from dying. Every available means that medical skill and science can suggest should be resorted to in order to rescue both lives from danger.

Morality teaches us that two wrongs do not make one right.

Any effect directly intended, no matter how good or legitimate, can never justify a means that is in itself wrong.

"FINIS NON JUSTIFICAT MEDIA."

I shall not presume to lay down before this learned body therapeutic measures whereby this desirable end can be obtained. It would be the height of temerity and presumption on my part to trespass on the threshold of medical and surgical authority, learning and science, and tell them what to do.

I have no credentials as either a medical teacher, a moralist, or a theologian.

I am however sustained by the fact, that an unknown quantity is sometimes used to confound the strong; and I have no hesitancy in proclaiming that a child, whether it be an embryo, a fetus, or a fully developed babe, has under all circumstances a legal and moral right to its life, and ought never to be deliberately killed.

Under no circumstances does a case ever call for an artificial abortion. It goes without saying that this rule does not apply to a dead fetus. Hence every pregnancy must be allowed to fill unmolested its natural term, or to progress in well defined and determined cases to the period of viability.

Craniotomy is another criminal and dangerous proceeding which ought to have professional condemnation. I cannot dwell on this subject, at the same time so vast and interesting, without transcending the reasonable limit and scope of this paper.

I can but reiterate the warning that cephalotripsy is no less wrong and criminal than revolting. The concurrent testimony of the leading gynecologists and abdominal surgeons of Europe and America is now decidedly in favor of Cæsarean operation. The possibility of saving the mother and child is not only being made more and more evident, but has almost reached the dignity of a certainty, owing to the gigantic strides of modern surgery. Cæsarean section is no longer dreaded except, perhaps, by the non-professionals, who from the remotest times, have been reared to shudder at the very thought of a delivery through ventral walls.

Modern surgery, with its anesthetics and antiseptic methods, bids a skillful and conscientious operator to adopt as an elective proceeding a method which inflicts less pain and requires less time than perforation and is likely to save two lives instead of one. When we think of such splendid results as have been achieved by Velpeau, Tait, Porro, Leopold, Saenger, Goodell, Cameron, Stone, Joseph Price, Wyeth, W. H. Parish, and many others, we cannot fail but to acknowledge that the day is fast approaching when the Cæsarean, the Porro operation, and other conservative procedures which offer to save two lives, will supersede the destroying of the child, that the chances of the mother's recovery may be secured.

The Porro method, inasmuch as it renders the mother sterile and saves her from the necessity of having to undergo again the Cæsarean section, is gaining favor with the profession, and is now much in vogue both in Europe and America. It can be performed quicker than the conservative operation, and the danger of the shock is not greater.

Dr. W. H. Parish says that, "Of one hundred women on whom Cæsarean section is performed under favorable conditions and with attainable skill, about ninety-five mothers should recover and fully the same number of children. Of one hundred craniotomies ninety-five mothers, or probably a larger number, will recover, and of course none of the children. The problem resolves itself into this: Which shall we choose? Cæsarean section, with one hundred and ninety beings as the result, or cranitomy with about ninety-five living beings? The conclusion seems inevitable that if the child can possibly live after birth, the performance of craniotomy can be justified no longer from any standpoint whatever."

I have written this paper in all candor and sincerity, and with no desire to wound the feelings of those who may differ with me on this most important and serious question. As a brother physician, I entertain none but the most vivid sentiments of kindness, respect, and admiration for every member of this noble association, as well as for all those who are honestly engaged in the praiseworthy task of relieving the sick and afflicted.

I do trust that whatever I have said shall not be so construed as to put my colleagues, who may have had recourse to some of the practices I have just denounced, on a level with abortion mongers, and unprincipled craniotomists.

My object is to remind a conscientious but erring brother of the fact that revelation and morality should have a preponderating voice in refraining him from taking an active and responsible part in the *slaughter of the innocents*.

